# Network inference with ensembles of bi-clustering trees

**DOI:** 10.1186/s12859-019-3104-y

**Published:** 2019-10-28

**Authors:** Konstantinos Pliakos, Celine Vens

**Affiliations:** 1 KU Leuven, Campus KULAK, Department of Public Health and Primary Care, Faculty of Medicine, Kortrijk, Belgium; 2 ITEC, imec research group at KU Leuven, Kortrijk, Belgium

**Keywords:** Biomedical networks, Network inference, Interaction prediction, Tree-ensembles, Multi-label classification

## Abstract

**Background:**

Network inference is crucial for biomedicine and systems biology. Biological entities and their associations are often modeled as interaction networks. Examples include drug protein interaction or gene regulatory networks. Studying and elucidating such networks can lead to the comprehension of complex biological processes. However, usually we have only partial knowledge of those networks and the experimental identification of all the existing associations between biological entities is very time consuming and particularly expensive. Many computational approaches have been proposed over the years for network inference, nonetheless, efficiency and accuracy are still persisting open problems. Here, we propose bi-clustering tree ensembles as a new machine learning method for network inference, extending the traditional tree-ensemble models to the global network setting. The proposed approach addresses the network inference problem as a multi-label classification task. More specifically, the nodes of a network (e.g., drugs or proteins in a drug-protein interaction network) are modelled as samples described by features (e.g., chemical structure similarities or protein sequence similarities). The labels in our setting represent the presence or absence of links connecting the nodes of the interaction network (e.g., drug-protein interactions in a drug-protein interaction network).

**Results:**

We extended traditional tree-ensemble methods, such as extremely randomized trees (ERT) and random forests (RF) to ensembles of bi-clustering trees, integrating background information from both node sets of a heterogeneous network into the same learning framework. We performed an empirical evaluation, comparing the proposed approach to currently used tree-ensemble based approaches as well as other approaches from the literature. We demonstrated the effectiveness of our approach in different interaction prediction (network inference) settings. For evaluation purposes, we used several benchmark datasets that represent drug-protein and gene regulatory networks. We also applied our proposed method to two versions of a chemical-protein association network extracted from the STITCH database, demonstrating the potential of our model in predicting non-reported interactions.

**Conclusions:**

Bi-clustering trees outperform existing tree-based strategies as well as machine learning methods based on other algorithms. Since our approach is based on tree-ensembles it inherits the advantages of tree-ensemble learning, such as handling of missing values, scalability and interpretability.

## Background

Network representations are ubiquitous in systems biology. They can be homogeneous, such as protein protein interaction ones, or heterogeneous, such as drug protein interaction or gene regulatory ones. The inference of those networks, a task often denoted as interaction prediction, is of fundamental importance. For example, drug-protein interaction (DPI) prediction has a substantial role in drug discovery or drug repositioning (i.e., the identification of new applications of already existing drugs) [1]. The analysis of DPI networks can provide vital information for the understanding of disease mechanisms and cell biochemical processes. *In silico* predictions of DPI leverage research in the pharmaceutical domain, accelerating drug development while diminishing the risk of failures [2]. Such failures are often extremely expensive, especially when they occur at a late stage of the drug discovery process. New interactions between candidate drugs and proteins others than their original targets can also reveal possible side effects of those drugs [3]. Moreover, the identification of new interactions between approved drugs and proteins contributes to drug repositioning, revealing new possible applications of already existing drugs. Furthermore, the deciphering of gene regulatory networks (GRN) is fundamental for making any progress in organism functioning and pathology understanding [4]. The mapping of the topology of those networks can potentially reveal the function of complex biological processes that take place in an organism and thereby improve diagnostics and prognostics.

Currently, we have only partial knowledge of those networks. Despite the effort made and the existing computational approaches for interaction prediction, there is definitely space for further improvement as accuracy and efficiency are still open problems. Therefore, there is need of new effective machine learning methods for network inference. Machine learning models are an incomparably useful guide for future in vitro or in vivo experiments and also reveal latent knowledge about biological networks. The latter is achieved by using interpretable models, such as decision tree-based ones.

Generally, machine learning has significantly contributed to systems biology and bioinformatics [5–8], providing the means to perform predictions and insightful data analysis. Supervised learning is the domain that has drawn the greatest attention. The learning models that fall in this category are built on an input set *X* and an output set *Y*. More precisely, the instances (e.g., genes, drugs, proteins) are described by input variables and are also associated with one or more output variables. These input variables are called features while the output ones targets or labels. The objective for a supervised learning method is to learn a function (*f*:*X*→*Y*) on the features of a training set of instances able to predict the output variable [9]. Following the inductive setup, as soon as the learning procedure is over, the function can be used to perform predictions for unseen instances. In cases where the output variable is numeric, the task is called regression while when it is categorical (i.e., prediction of a class), the task is called classification. In cases where multiple output variables need to be predicted instead of a single one the task is denoted as multi-output (multi-target) prediction [10]. Multi-target prediction is divided in multi-target classification (i.e., the targets have nominal values) or multi-target regression [11]. In addition, there is another case which is known as multi-label classification [12, 13]. Multi-label classification can be characterized as a multi-target regression task where one has only binary target values, or as a multi-target classification task, having only two classes (0 and 1). Here, we focus on multi-label classification and thereby refer to the output variables as labels.

**Fig. 1:**
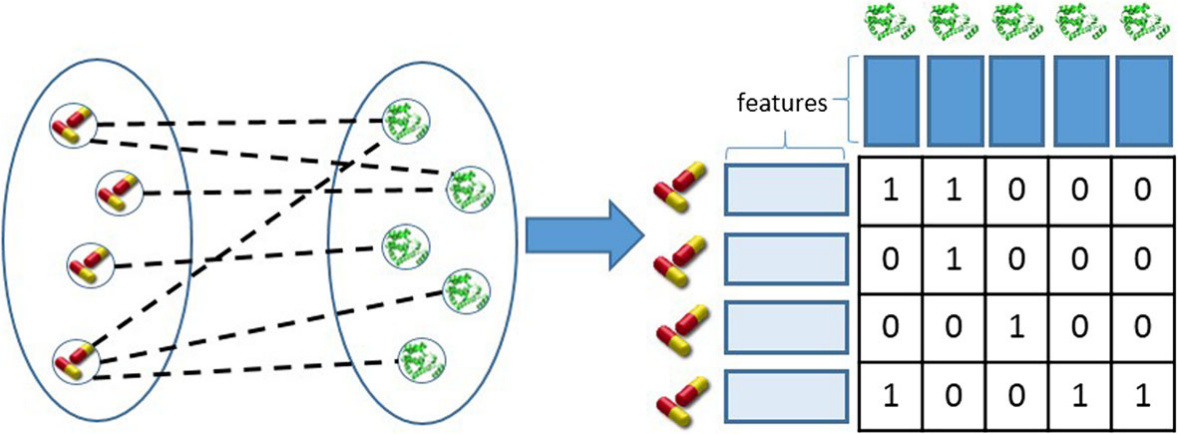
Illustration of a (bi-partite) DPI interaction network

A heterogeneous network (e.g., a drug-protein interaction network) can be formulated as a collection of two sets of items that interact with each other. Each item set is described by its own features. Those features compose the background information in our problem. For example, in a drug-protein interaction network the two item sets are the drugs, described by chemical structure similarities, and target proteins described by protein sequence similarities. The interactions are the links connecting the nodes of the network and are often represented as a matrix. In Fig. 1, an example of such a network setting is displayed.

There are mainly two approaches to apply a learning method in this framework: the local approach [14] and the global one [15]. Based on the local approach, one first decomposes the data into different (traditional) feature sets, solves each set’s learning task separately, and integrates the results. Following the global approach, the learning method is adjusted in order to handle the structured representation directly. A discussion of the two aforementioned approaches takes place in [16].

In this paper, we handle network inference as a multi-label classification task, integrating background information (i.e., features) from both item sets in the same network framework. The method proposed here is a global approach, extending multi-output decision tree learning to the interaction data framework. More specifically, we propose a tree-ensemble based approach extending the decision tree-based method proposed in [17]. Each tree of the ensembles is built considering split candidates in both row and column features and thereby partitions the interaction matrix both row-wise and column-wise. A traditional multi-output tree partitions the interaction matrix only row-wise (clustering). However, our approach introduces also column-wise partitioning, providing thereby a bi-clustering of the interaction matrix. This way, we refer to the proposed method as *ensembles of bi-clustering trees*. Moreover, we performed a thorough comparison study, including traditional global and local tree-ensemble approaches. Our comparison study complements a previous one [18], introducing *ensembles of bi-clustering trees* to the group of tree-ensemble learning approaches for network inference. For our comparison study, we employed the extremely randomized trees (ERT) [19] and random forests (RF) [20]. These two ensemble methods are well established and also powerful. We discuss differences between the ERT-based and RF-based methods in our setting. Next, we extended our evaluation study by comparing our approach against effective (not tree-ensemble based) network inference methods from the literature. For evaluation purposes, we employed several heterogeneous interaction networks, which are publicly available and act as benchmark datasets in the field. The obtained results demonstrate the merits of our proposed learning method. In addition to that, we performed experiments on two versions (v3.1, v4) of the chemical-protein interaction database STITCH. We trained our proposed model using v3.1 and tested it on v4. The performance and application importance of our model was reaffirmed, as we managed to predict non-reported interactions in v3.1 that appeared later in v4.

### Related work

Machine learning has been broadly applied to network inference [4],[21]. Several approaches were based on matrix factorization [21, 22]. Network inference was handled as a prediction task on DTI networks in [23], where multiple-kernel learning was used, and [24], where random walk with restart was employed. In [25], the authors computed drug-based, target-based, and network topology-based kernels, addressing next the DTI prediction task employing the regularized least squares classifier. This approach was extended in [26] to achieve predictions for new candidate drugs or target proteins. A semi-supervised method for DTI prediction was proposed in [27]. Similarities between drugs and between targets were computed and used as input for a robust PCA model. In [28], drug-target interaction (DTI) prediction was pursued using only network topology information. They computed similarities between the nodes of a DTI network based only on the network structure. In [18], the authors addressed the problem of network inference as a supervised learning task. They specifically used ERT performing a comparison study between three different learning strategies and discussed the corresponding benefits and drawbacks. The multi-label k-nearest neighbor (MLkNN) [29] classifier was used in [30] and [31]. Specifically, in [30], the authors applied clustering on the targets corresponding features building a second interaction matrix. They referred to this strategy as super-target clustering. They applied MLkNN on both matrices separately and combined the predictions. In [31], a drug side effect prediction method was proposed where the authors integrated information from multiple sources and built individual feature-based predictors. Furthermore, in [32], a re-ranking gene regulatory network inference strategy was proposed as a post processing approach that could be combined with any supervised or unsupervised method.

Many methods also used graph embedding and feature extraction mechanisms boosting the performance of predictors such as random forest or neural networks. In [33], the authors investigated how graph embedding algorithms contribute to link prediction in biomedical networks. In [34], a feature set was extracted using graph mining and then a random forest classifier was applied to predict interactions. Similarly in [35], the topology of the DTI network was exploited for feature extraction. The final predictions were the output of a random forest classifier.

Many studies were presented showing that methods which combine the outputs of multiple algorithms in a consensus setting are very effective. Targeting at gene regulatory network (GRN) inference (reconstruction), a synergistic strategy enlisting about thirty methods was presented in [36]. Furthermore, a semi-supervised approach which combines the predictions made by multiple inference approaches was proposed in [37]. In that work, the consensus-based method combined the prediction of the employed network inference algorithms in a multi-view setting. Ezzat et al. [38] also tackled DTI prediction with ensemble learning in a class imbalance aware strategy. In [39], predictions by several methods were used and integrated into a learning to rank strategy.

Publicly available chemical and biological databases, such as STRING [40], ChEMBL [41], Gene Ontology [42], KEGG [43], UniProt [44], DrugBank [45], and STITCH [46] are crucial for the development of the aforementioned computational methods. These databases store vital information and act as sources for the development of modern machine learning methods.

All the aforementioned methods achieved a fair predictive performance. Nevertheless, there is still much space for improvement, especially considering the complexity of the network inference task. There are many types of networks (e.g., metabolic, drug-target, gene regulatory networks) and often methods that are focused on one specific type, for example DTI networks, are not necessarily effective when transferred to another type of network. Here, we propose a broad method that is not restricted to a specific network type. Moreover, several approaches proposed over the years (some of them described above) can be applied only in a transductive strategy [47]. This means that the test instances are required during the training of the algorithms. Here, we focus on inductive models, where the prediction model is built during the training process and then it can perform predictions for new data. Our approach is also based on tree-ensembles inheriting thereby the advantages of tree-ensemble learning, such as handling of missing values, scalability and interpretability. Besides predictive accuracy, the proposed approach also provides an interpretable bi-clustering.

## Method

In this section, first a broad view of tree-ensemble learning and multi-label classification is given. Next, we discuss the problem of network inference and traditional tree-ensemble approaches applied to it. Finally, our proposed method is presented.

### Multi-output tree-ensembles

Decision tree induction algorithms [48] follow a top-down induction method. The top node is denoted as the root and it contains the complete training set. The nodes are recursively split based on a split-test that is applied to one of the features that describe the instances. The optimal split features and their corresponding split points are selected based on a split quality criterion (e.g., entropy, variance reduction etc.). The tree growing procedure stops when the data contained in a node is pure w.r.t. the labels, or when another stopping criterion holds. Then the node is called a leaf and a label is assigned to it. When it comes to unseen instances, the labels are obtained by letting the instances traverse the tree ending up in a leaf node.

The predictive performance of decision trees is particularly boosted when they are combined with ensemble methods [20], providing often state-of-the-art results. Ensembles of trees also cure the unwanted overfitting effect and are known as more stable models than single tree-based ones. Two of the most popular tree-ensemble approaches are the random forests (RF) [20] and the extremely randomized trees (ERT) [19]. The RF uses bootstrap replicates of the training set and random selection of the features describing the samples. More specifically, each decision tree of the ensemble is constructed on a random subset of the training set. Every node of that tree is split by computing the best possible split among a random subset of *Λ* selected feature candidates. The final prediction is yielded as the average of the predictions of individual trees. The ERT is an extension of RF which omits bootstrapping and splits every node by selecting the best possible split from *Λ* random ones. Ensembles of trees are not so easily interpreted as single trees though. However, there are strategies [49] that can transform an ensemble of trees to a single tree, preserving therefore the interpretability value. Tree-ensembles also provide a natural feature ranking, evaluating this way the contribution of each feature to the learning process.

Apart from their extension to ensembles, tree-based models have also been extended towards multi-output tasks, such as multi-label classification [11, 12]. In a multi-label classification task, for each instance (e.g., protein) the set of labels (e.g., interactions) is represented as a vector of size equal to the total number of labels. Then, the possible splits are evaluated by calculating the variance reduction over these vectors, instead of over single values. Next, the average of the target vectors of the instances that are present in a leaf is computed. Once the model has been built, it can be used for prediction of new (unseen) instances.

### Interaction network inference

Let *G* define a heterogeneous network with two finite sets of nodes *N*={*n*_1_,⋯,*n*_|*N*|_} and *M*={*m*_1_,⋯,*m*_|*M*|_}. Each node of the network corresponds to a biological entity (e.g, drug, gene, protein) and is described by a feature vector. The links connecting the nodes of the network represent interactions between the corresponding biological entities (e.g., drug-protein interactions). The set of existing or not existing links of the network are formulated as an interaction matrix $\mathbf {Y} \in \mathfrak {R}^{|N|\times |M|}$. Every item *y*(*i*,*j*)∈**Y** is equal to 1 if an interaction between items *n*_*i*_ and *m*_*j*_ holds and 0 otherwise. Networks that are homogeneous, such as protein-protein interaction ones, have two identical sets of nodes (i.e., *N*=*M*) and consist a particular case of the broader framework described above.

Network inference can be treated in a supervised learning manner and particularly as a classification task on pairs of nodes. Specifically, the goal is to build a model that receives pairs of network nodes as input and outputs a probability that an interaction between these two nodes exists. Focusing on the inductive setup, the learning model is built on a training set of interacting or non-interacting pairs of nodes. After the learning model is built, it can be used to perform predictions for unseen pairs.

**Fig. 2:**
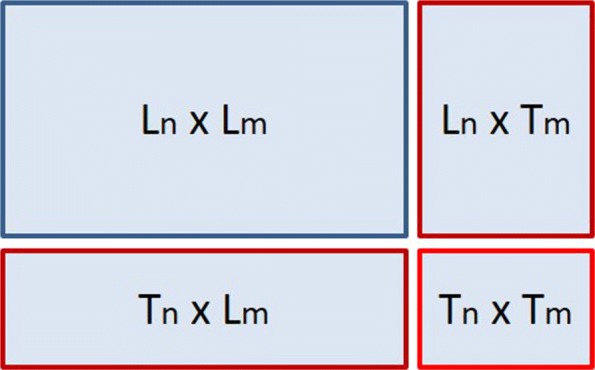
The prediction setting of an interaction network
- Learned rows - Test columns (*L*_*n*_×*T*_*m*_): predictions regarding row instances that have been included in the learning procedure and unknown (new) column instances.
- Test rows - Test columns (*T*_*n*_×*T*_*m*_): predictions regarding unknown (new) row instances and unknown (new) column instances.

The prediction of the interactions in networks is not as straight-forward as in traditional classification tasks where one has a single set of instances. When it comes to networks, one can perform predictions where the test is a pair of unknown instances (e.g., drugs, proteins, genes) and predictions where one of two instances is included in the learning procedure. Predicting pairs of unknown instances is a greatly more difficult task. In particular, the prediction framework of our problem is displayed in Fig. 2 [17]. The (*L*_*n*_×*L*_*m*_) corresponds to the interaction matrix (i.e., **Y**) which we assume is available during the training process. As one considers supervised learning, the mining setting can be divided into 3 sub-settings.

- Test rows - Learned columns (*T*_*n*_×*L*_*m*_): predictions regarding unknown (new) row instances and column instances that have been included in the learning procedure.

### Traditional tree-ensembles for network inference

As mentioned in the introduction, there are two approaches to apply a learning technique in the network framework, the local approach [14] and the global one [15]. Let $\mathbf {X_{n}} \in \mathfrak {R}^{|N|\times |D_{n}|}$ be the representation of the *N* set of nodes and $\mathbf {X_{m}} \in \mathfrak {R}^{|M|\times |D_{m}|}$ be the representation of the *M* set of nodes.

In the local approach, one multi-output classifier is built over nodes *N* and another multi-output classifier is built over nodes *M*. The outputs of the two classifiers are integrated yielding the final predictions.

**Fig. 3:**
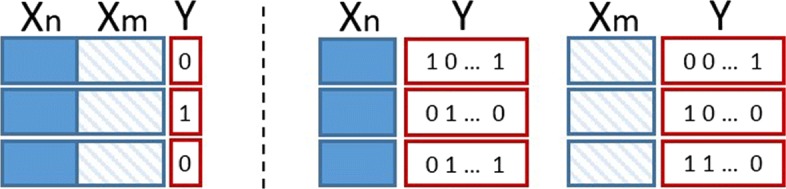
A description of the two learning approaches. Left the global single output and right the local multiple output approach

In the global approach, only one classifier is built, incorporating the two interactive sets in a unified framework. Traditionally, a single-output classifier is built over the Cartesian product of the two sets of nodes, $\mathbf {X_{g}} \in \mathfrak {R}^{(|N|*|M|)\times (|D_{n}|+|D_{m}|)}$. In Fig. 3, a representation of the two settings is illustrated.

### Ensembles of bi-clustering trees

**Fig. 4:**
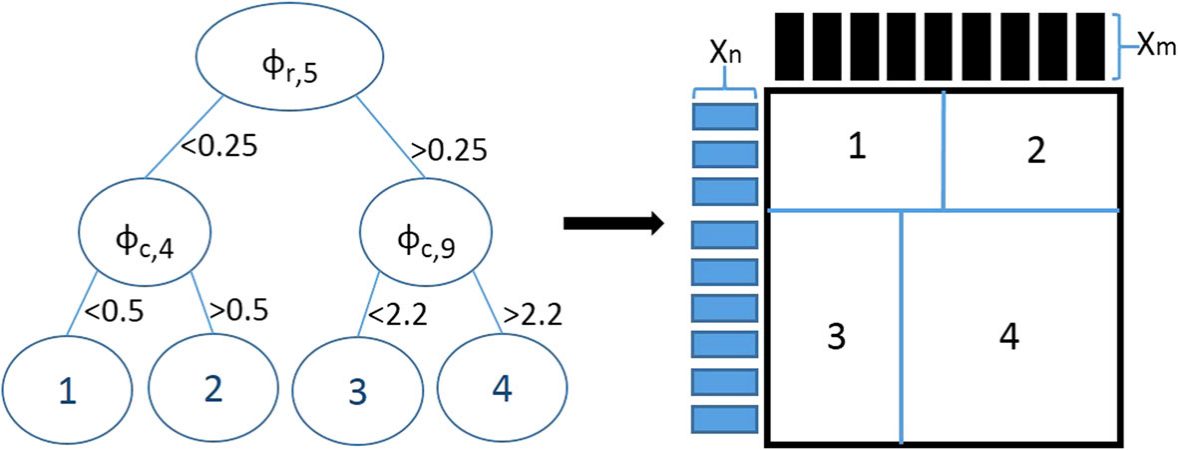
Illustration of a bi-clustering tree along with the corresponding interaction matrix that is partitioned by that tree. Let *ϕ*_*r*_ and *ϕ*_*c*_ be the features of the row and column instances respectively

A multi-label driven extension of single decision trees for interaction prediction was presented in [17]. Here, we present the ensemble extension of our previous model. The input of our model consists of pairs of instances and the task is to predict a value of interest that is related to it. The bi-clustering inferred by a single tree is illustrated in Fig. 4 [17]. We originally build our model in the ERT setting but other ensemble strategies, such as RF, can be also applied. An important element in RF is the bootstrapping. In a global network setting one can perform bootstrapping on the samples that correspond to the rows of the interaction matrix, the columns, both rows and columns (blocks), or specific elements. Each tree in our ensemble grows considering as split-candidates for every node a random sub-set of both row and column features (i.e., features associated with the two instance sets) and therefore splitting the interaction (label) matrix both horizontally and vertically. The optimal split is picked aiming to maximize impurity (*Var*) reduction on interaction matrix **Y**, following the split selection strategy of ERT. In every node of the tree, when the split test is on a feature that corresponds to a row instance (e.g., a drug) then $Var=\sum _{j}^{M} Var(\mathbf {Y}_{j})$. When the split test is on a feature that corresponds to a column instance (e.g., a target protein) then $Var=\sum _{i}^{N} Var(\mathbf {Y}^{T}_{i})$, where *M*, *N*, and **Y**^*T*^ are the number of column instances, row instances, and the transpose matrix of **Y**, respectively. The partitioning of the interaction (label) matrix both horizontally and vertically deducts a bi-clustering [50] of the network. Each tree of the ensemble yields predictions that are averaged to generate the final predictions.

**Fig. 5:**
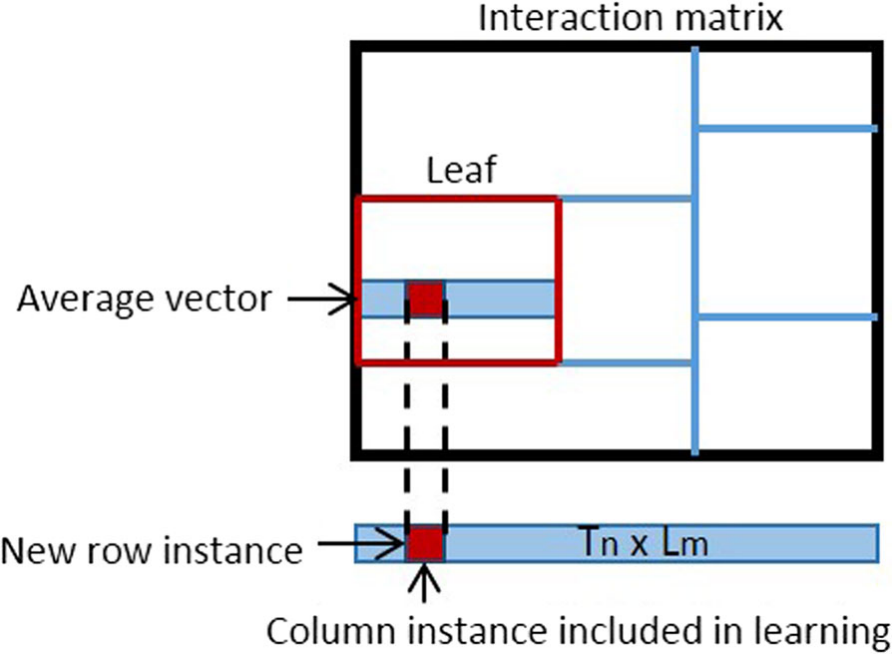
Illustration of the labeling strategy that is followed. Prediction of an interaction between a new row instance and a column instance included in learning

An important part of the tree-ensemble learning process is how to assign labels to the tree leaves. This is also known as the prototype function. In traditional trees the prototype function considers the majority class assigned to the training instances present in the leaf for classification, or the average of their target values for regression. The prediction for test instances is obtained by sorting them through the tree into a leaf node. In our bi-clustering tree method the prototype function differentiates the prediction returned in the leaves based on the prediction context. The followed labeling strategy is displayed in Fig. 5 [17]. More specifically, in *T*_*n*_×*L*_*m*_ the submatrix corresponding to the leaf is averaged vertically, generating a label vector **W** while in *L*_*n*_×*T*_*m*_ horizontally, generating a label vector **W**^*T*^. For *T*_*n*_×*T*_*m*_, the strategy of averaging all values in a leaf is followed. When it comes to new data and more specifically pairs of instances where the row-instance *n*_*i*_∉*L*_*n*_ and the column instance *m*_*j*_∈*L*_*m*_, one can be certain that the new pair will end up in a leaf (partition of the interaction matrix) that is associated with the *m*_*j*_∈*L*_*m*_. Then, the yielded prediction for the pair is the *w*_*k*_∈**W** that corresponds to *m*_*j*_. However, in tree-ensemble strategies such as random forests that adopt bootstrapping, this specific labeling mechanism can not hold as the column instance *m*_*j*_∈*L*_*m*_ may belong to the out-of-bag instances. What we propose thereby in such cases is to ignore bootstrapping in the construction of the prototype function. This means that bootstrapping can be used for the growing of the trees but then the whole training set should be used in the computation of the prototype function.

## Data

We first employed 6 datasets [18], that represent heterogeneous interaction networks. These are publicly available benchmark datasets that are often used in related studies. The interactions in those datasets are represented as binary values. Moreover, we extracted a subset of the STITCH database [46] in two versions (v3.1, v4) in order to validate the performance of the proposed approach. The summary of the datasets and their characteristics is shown in Table 1. It contains the number of row instances, column instances, and their corresponding feature sizes. Information about the number and proportion of existing interactions in each network is also disclosed.

**Table 1:** The datasets used in the evaluation procedure

*Dataset*	|*N*|×|*M*|	|*Features*|	|*interactions*|
ERN	1164×154	445−445	3293/179256 (1.8*%*)
SRN	1821×113	1685−1685	3663/205773 (1.7*%*)
DPI-E	664×445	664−445	2926/295480 (1*%*)
DPI-IC	204×210	204−210	1476/42840 (3.4*%*)
DPI-GR	95×223	95−223	635/21185 (3*%*)
DPI-NR	26×54	26−54	90/1404 (6.4*%*)
CPIv3.1	2154×2458	2154−2458	138513/5294532 (2.6*%*)
CPIv4	2154×2458	2154−2458	258618/5294532 (4.9*%*)

In particular:

- **E. coli regulatory network (ERN)** [51]. This heterogeneous network consists of 179256 pairs of 154 transcription factors (TF) and 1164 genes of E. coli (154×1164=179256). The feature vectors that represent the two sets consist of 445 expression values.
- **S. cerevisiae regulatory network (SRN)** [52]. This heterogeneous network is composed by interactions between TFs and their target S. cerevisiae genes. It is composed of 205773 pairs of 1821 genes and 113 TFs. The input features are 1685 expression values.
- **Drug–protein interaction networks (DPI)** [53]. The datasets in [53] correspond to 4 drug-protein interaction networks where the interactions between drugs and target proteins are represented as binary values. The target proteins correspond to 4 pharmaceutically useful categories: nuclear receptors (NR), G-protein-coupled receptors (GR), ion channels (IC), and enzymes (E). The drugs related features are the similarities of their chemical structure. The feature vectors associated with the target proteins consist of similarities based on the alignment of protein sequences. Those sequence similarities were measured using the normalized Smith-Waterman score.
- **Compound–protein association network**. We extracted another dataset that corresponds to a chemical–protein interaction (CPI) network (human) from the STITCH database [46]. In particular, we extracted two datasets corresponding to the same network, as it appears in versions 3.1 and v4 of the STITCH database. Interactions in STITCH are derived from lab experiments, knowledge in manually curated databases, text mining techniques applied to literature, and computational predictions. The cumulative scores that correspond to whether an interaction between two nodes exists is depicted in range from 0 to 1. Here, we have converted these numeric values to binary, setting to 1 all the non-zero values. We filtered the database based on frequency of interactions, extracting only a subset of 2154 compounds and 2458 proteins. We extracted characteristics for both chemical compounds and proteins and used them as features to learn our model. The input feature vectors for proteins represent the similarity with all proteins in terms of sequence. The similarities between the proteins were computed as $s(x_{pi},x_{pj}) = \frac {sim(x_{pi},x_{pj})}{\sqrt {|x_{pi}|} * \sqrt {|x_{pj}|}}$, where *sim*(*x*_*pi*_,*x*_*pj*_) is the pairwise global alignment score between sequences *x*_*pi*_ and *x*_*pj*_. The input feature vectors for chemicals represent the similarity with all chemicals in terms of their structure. After collecting the SMILES strings of the chemical compounds present in our dataset we generated corresponding FP2 fingerprints using Open Babel [54], an open source cheminformatics toolbox. Next, we computed compound similarities as $s(x_{i},x_{j}) = \frac {|x_{i} \cap x_{j}|}{|x_{i} \cup x_{j}|}$.

## Results

### Evaluation metrics

The metrics that were used are the area under precision recall curve (AUPR) and the area under the receiver operating characteristic curve (AUROC). A PR curve is defined as the Precision ($\frac {TP}{TP+FP}$) against the Recall ($\frac {TP}{TP+FN}$) at various thresholds. A ROC curve is defined as the true positive rate ($\frac {TP}{TP+FN}$) against the false positive rate ($\frac {FP}{FP+TN}$) at various thresholds. The true-positive rate is equal to recall. True-positive rate is also denoted as sensitivity while false-positive rate is also denoted as (1 - specificity). The aforementioned measures were employed in a micro-average setup.

A common attribute of biomedical interaction networks is the presence of sparsity. As reflected in Table 1, the existing interactions average around 3%. This means that only 3% of the labels (i.e., items of the interaction matrix) are equal to 1 and the rest 97% are equal to 0. The corresponding classification task is therefore particularly imbalanced. It has been shown that AUPR is more informative than AUROC when it comes to highly imbalanced classification problems [55, 56]. This is based on that AUROC rewards true negative predictions (leading to a low false positive rate), which are easy to obtain in very sparse datasets, whereas AUPR focuses on recognizing the positive labels. The employment of AUPR and AUROC in biomedical networks was also investigated in [57].

### Evaluation protocol

We start our evaluation study by comparing the ensemble of bi-clustering trees (eBICT) to the two traditional tree-ensemble approaches used for interaction prediction in networks, namely global single output (GLSO) and local multiple-output (LOCMO) [18]. Afterwards, we compare eBICT to two powerful methods in DTI prediction. Although we have initially developed our model in the extremely randomized trees (ERT) setting we also compare our bi-clustering tree approach in a random forests (RF) setting for completeness. All methods were validated in terms of predictive performance. The methods are compared in all three prediction settings (i.e., *T*_*n*_×*L*_*m*_, *L*_*n*_×*T*_*m*_, and *T*_*n*_×*T*_*m*_). The comparison was performed independently for every setting.

In *T*_*n*_×*L*_*m*_ and *L*_*n*_×*T*_*m*_ a 10-fold cross validation (*CV*) setting on nodes (i.e., CV on row instances and CV on column instances of the network, respectively) was applied. In *T*_*n*_×*T*_*m*_, a *CV* setting on blocks of row and column instances was applied, excluding one row fold and one column fold from the learning set, and using their combined interactions as test set. Due to the sparsity of the data, 10-fold *CV* in *T*_*n*_×*T*_*m*_ was burdensome as there were folds containing only zeros and thereby a 5-fold *CV* setting over blocks of row and column instances (i.e., 5×5=25 folds) was employed. For all settings and tree-ensemble algorithms 100 trees were used and no tree-pruning was applied.

### Comparison results

The compared tree-ensemble methods, eBICT, GLSO and LOCMO, were first evaluated in an ERT ensemble strategy and the results are presented in Table 2. As it can be observed, eBICT outperforms the compared models in most cases. More specifically, eBICT demonstrates overall superior predictive performance in terms of AUPR in all settings and slightly inferior AUROC results only in *L*_*n*_×*T*_*m*_ and *T*_*n*_×*L*_*m*_. We next evaluated the proposed approach in a RF ensemble setting. When it comes to bootstrapping, we applied bootstrapping on instances corresponding to both rows and columns of the interaction matrix. As reflected in Table 3, eBiCT outperforms both GLSO and LOCMO in terms of AUPR in all three prediction settings. The AUROC results obtained by eBICT are inferior in *L*_*n*_×*T*_*m*_ and *T*_*n*_×*L*_*m*_. However, it should be highlighted that AUPR is more informative than AUROC when it comes to highly imbalanced classification problems [55–57].

**Table 2:** AUPR and AUROC results for the compared methods. The tree-ensemble setting is the ERT

AUPR	*T*_*n*_×*L*_*m*_			*L*_*n*_×*T*_*m*_			*T*_*n*_×*T*_*m*_		
**Data**	*eBICT*	*GLSO*	*LOCMO*	*eBICT*	*GLSO*	*LOCMO*	*eBICT*	*GLSO*	*LOCMO*
ern	0.397	0.397	0.404	0.043	0.041	0.043	0.048	0.047	0.035
dpie	0.645	0.638	0.626	0.303	0.294	0.309	0.175	0.163	0.179
dpii	0.544	0.535	0.541	0.327	0.326	0.33	0.073	0.07	0.074
dpig	0.239	0.24	0.234	0.345	0.329	0.318	0.084	0.083	0.073
dpin	0.385	0.362	0.395	0.507	0.506	0.513	0.106	0.105	0.106
srn	0.157	0.158	0.17	0.028	0.03	0.028	0.022	0.024	0.018
Avg	**0.395**	0.388	**0.395**	**0.259**	0.254	0.257	**0.085**	0.082	0.081
AUROC	*T*_*n*_×*L*_*m*_			*L*_*n*_×*T*_*m*_			*T*_*n*_×*T*_*m*_		
ern	0.845	0.849	0.856	0.603	0.594	0.602	0.729	0.721	0.645
dpie	0.873	0.865	0.87	0.825	0.835	0.815	0.719	0.713	0.684
dpii	0.824	0.82	0.824	0.793	0.789	0.8	0.582	0.566	0.54
dpig	0.662	0.654	0.659	0.854	0.85	0.848	0.655	0.658	0.601
dpin	0.625	0.61	0.614	0.786	0.777	0.78	0.578	0.572	0.535
srn	0.794	0.796	0.807	0.544	0.54	0.532	0.551	0.568	0.497
Avg	0.771	0.766	**0.772**	0.734	0.731	**0.735**	**0.636**	0.633	0.584

Best values appear in boldface

**Table 3:** AUPR and AUROC results for the compared methods. The tree-ensemble setting is the RF

AUPR	*T*_*n*_×*L*_*m*_			*L*_*n*_×*T*_*m*_			*T*_*n*_×*T*_*m*_		
**Data**	*eBICT*	*GLSO*	*LOCMO*	*eBICT*	*GLSO*	*LOCMO*	*eBICT*	*GLSO*	*LOCMO*
ern	0.399	0.386	0.404	0.049	0.047	0.055	0.065	0.052	0.052
dpie	0.613	0.607	0.6	0.32	0.302	0.323	0.175	0.155	0.167
dpii	0.518	0.5	0.496	0.341	0.324	0.342	0.065	0.068	0.07
dpig	0.233	0.226	0.219	0.35	0.318	0.329	0.085	0.077	0.069
dpin	0.39	0.333	0.367	0.502	0.481	0.495	0.105	0.1	0.095
srn	0.149	0.133	0.168	0.028	0.032	0.025	0.023	0.023	0.018
Avg	**0.384**	0.364	0.376	**0.265**	0.251	0.262	**0.086**	0.079	0.079
AUROC	*T*_*n*_×*L*_*m*_			*L*_*n*_×*T*_*m*_			*T*_*n*_×*T*_*m*_		
ern	0.836	0.846	0.857	0.602	0.645	0.61	0.763	0.732	0.642
dpie	0.831	0.87	0.868	0.819	0.826	0.819	0.736	0.712	0.675
dpii	0.792	0.817	0.814	0.808	0.799	0.801	0.579	0.573	0.529
dpig	0.574	0.692	0.655	0.853	0.863	0.855	0.639	0.641	0.589
dpin	0.511	0.661	0.583	0.75	0.775	0.774	0.59	0.567	0.505
srn	0.812	0.779	0.806	0.518	0.569	0.532	0.558	0.558	0.496
Avg	0.726	**0.778**	0.764	0.725	**0.746**	0.732	**0.644**	0.631	0.573

Best values appear in boldface

Furthermore, it should be highlighted that both ERT-based and RF-based eBICT performs better than its competitors in the most difficult task of predicting interactions between pairs of totally unseen instances (i.e., *T*_*n*_×*T*_*m*_). Apart from predictive performance, eBICT is better applicable on *T*_*n*_×*T*_*m*_ than *LOCMO*. eBICT is trained over *L*_*n*_×*L*_*m*_ and it can perform predictions for all three settings directly. On the contrary, as pointed out in [17], every time an unseen pair of instances arrives (i.e., *T*_*n*_×*T*_*m*_) *LOCMO* has to train two new models, posing a serious disadvantage to the on-line application of *LOCMO* as well as other local approaches following the same strategy.

### Comparison with other approaches from literature

Although we focus on tree-ensemble learning, we extended our evaluation study by comparing our approach against two effective network inference methods from the literature. More specifically, we compared eBICT against [26] and [30] following the same strategy as above. Both [26] and [30] were originally proposed for inferring DTI networks. The method in [26] is denoted as BLM-NII and is a kernel-based local approach. Here, we used the rbf kernel as proposed in the original paper and selected the linear combination weight (*α* parameter) from a range of {0.1,0.25,0.5,0.75,1.0,1.25,1.5} through a 5-fold CV inner tuning process. The method in [30] is denoted as super target clustering (STC). It uses MLkNN in a target clustering-driven strategy. The optimal number of nearest neighbors in STC was selected from a range of {3,5,7,9,11} through 5-fold CV inner tuning.

The obtained AUPR and AUROC results are presented in Table 4. It is shown that eBICT outperforms the compared approaches in terms of both AUPR and AUROC, reaffirming thereby its effectiveness.

**Table 4:** AUPR and AUROC results for the compared methods

AUPR	*T*_*n*_×*L*_*m*_			*L*_*n*_×*T*_*m*_			*T*_*n*_×*T*_*m*_		
**Data**	*eBICT*	*BLM*−*NII*	*STC*	*eBICT*	*BLM*−*NII*	*STC*	*eBICT*	*BLM*−*NII*	*STC*
ern	0.397	0.401	0.378	0.043	0.03	0.032	0.048	0.029	0.029
dpie	0.645	0.489	0.635	0.303	0.217	0.233	0.175	0.047	0.122
dpii	0.544	0.338	0.542	0.327	0.245	0.294	0.073	0.075	0.054
dpig	0.239	0.168	0.197	0.345	0.277	0.294	0.084	0.033	0.06
dpin	0.385	0.373	0.351	0.507	0.476	0.48	0.106	0.079	0.06
srn	0.157	0.126	0.133	0.028	0.032	0.032	0.022	0.019	0.02
Avg	**0.395**	0.316	0.373	**0.259**	0.213	0.228	**0.085**	0.047	0.058
AUROC	*T*_*n*_×*L*_*m*_			*L*_*n*_×*T*_*m*_			*T*_*n*_×*T*_*m*_		
ern	0.845	0.861	0.842	0.603	0.552	0.549	0.729	0.579	0.571
dpie	0.873	0.832	0.823	0.825	0.823	0.729	0.719	0.571	0.602
dpii	0.824	0.749	0.773	0.793	0.777	0.767	0.582	0.569	0.533
dpig	0.662	0.527	0.528	0.854	0.815	0.835	0.655	0.472	0.508
dpin	0.625	0.622	0.553	0.786	0.8	0.807	0.578	0.532	0.423
srn	0.794	0.832	0.8	0.544	0.532	0.505	0.551	0.493	0.518
Avg	**0.771**	0.737	0.72	**0.734**	0.717	0.699	**0.636**	0.536	0.526

Best values appear in boldface

### Predicting associations between compounds and proteins

We also investigated the performance of eBICT by extracting a subset of the chemical compound association database STITCH. More specifically, we employed the specific dataset in two versions. The first derives from STITCH v3.1 and the second from STITCH v4. There are many links in the compound protein network that are not reported in v3.1 but exist in v4. We train our method using the interaction matrix that corresponds to v3.1 and evaluate the predictions using the matrix of v4. The purpose of this experiment is to investigate whether the application of the proposed learning approach and more specifically the inferred bi-clustering can reveal not-yet-reported associations between existing nodes of a network (i.e., *L*_*n*_×*L*_*m*_ setting).

As in *T*_*n*_×*L*_*m*_ and *L*_*n*_×*T*_*m*_ settings the multi-label structure of the matrix was preserved both in the tree-growing step and leaf-labelling step of the learning process. The experiment in detail was as follows: First, we trained eBICT in v3.1 and re-labelled the interactions between the existing nodes based on the inferred bi-clustering. This can be interpreted as performing predictions for the training set. Next, we compare the new labels to the labels of v4, investigating to what extent newly identified node associations are reported in the more recent version of the same database (v4). Here, as we focus on identifying non-reported interactions, we measure the links originally labeled as 0 in v3.1. These links can be either 0 or 1 in v4. Specifically, 3.5*%* of the links that are 0 in v3.1 appear as non-zero in v4.

First we measure the prediction (re-labeling) performance in terms of AUROC and AUPR and then we precisely check the top 20 associations identified by our method. Note that the proposed approach outputs a probability and not just binary values, therefore those top associations correspond to the links with the highest probability. More precisely, this set of 20 top predicted associations corresponds to a probability threshold of 0.65 in our algorithm. The experiment yielded an AUROC value equal to 0.626 and an AUPR equal to 0.079. It is interesting to observe that all our top 20 predicted associations were present in v4. As explained above, those associations were not reported in v3.1 (labelled as 0).

Another interesting point is that originally STITCH provides non-binary interaction data. The interaction scores in STITCH are in a range between 0 and 1. The scores stem from lab experiments, information from manually curated databases and computational approaches such as text mining. Thus, not all of those predicted associations can be translated into true molecular interactions. We also repeated the same experiment taking into account the actual scores in the STITCH database. In more detail, we trained eBICT based on numeric scores of v3.1. This way the problem can be interpreted as a more general multi-target regression task. The pair trifluoperazine and calmodulin-3 (not reported in v3.1) appears as the most probable compound protein association. The score of this pair in STITCH v4 is 0.907. This prediction can be also verified by searching through STITCH v5 and Drugbank where hard evidence is present (i.e., evidence stemming from manually curated databases). The full set of the 20 predicted associations is included as supplemental material [see Additional file 1].

## Discussion

In this paper we presented a novel tree-ensemble strategy to address the problem of network inference which is also known as interaction prediction or link prediction. We built our method, ensemble of bi-clustering trees (eBICT), upon our former approach presented in [17]. eBICT successfully transfers the traditional tree-ensemble learning setting, such as extremely randomized trees or random forests to the global network setting. Network inference is treated as a multi-label classification task, or more generally a multi-target prediction task, where different from the traditional setting, the labels are also characterized by features. In eBICT the tree-models are built on both instance and label corresponding features, partitioning thereby the interaction matrix (label space) both row-wise and column-wise. Thus, eBICT provides also an interpretable bi-clustering along with interaction prediction.

The work presented here focuses on interaction prediction and therefore a thorough comparison analysis between bi-clustering techniques would fall out of the scope of the specific study. The proposed method was compared against other tree-ensemble based network inference strategies which act as direct competitors. We also compared the proposed method against powerful (not tree-ensemble based) network inference approaches from the literature.

Throughout the recent years, many network inference methods were proposed. The majority was based on either synergistic learning strategies, where several classifiers were applied on the data and their outputs were aggregated to yield the final predictions, or feature extraction methodologies, where graph mining and other embedding methods were applied to extract new features that subsequently boosted the performance of common classifiers. It has to be highlighted that this kind of network inference methods are not considered as competitors to our method. On the contrary, eBICT can be applied in combination with the aforementioned approaches. For example, eBICT can be added to the models employed by a synergistic approach or it can be boosted by feature extraction techniques, replacing common models (e.g., RF) which are usually used.

Finally, we evaluated eBICT in different prediction settings, using both benchmark network datasets and an extracted compound protein association network. The obtained results affirmed the effectiveness of the proposed method. As eBICT is a tree-ensemble method, it adopts all the advantages of decision tree based learning. It is scalable, computationally efficient, interpretable, and capable of handling missing values. In contrast to the majority of methods developed for network inference, our method is also an inductive approach, which means that after the training process is over, the predictive function which has been built, can be used to perform predictions for new data. This way, no re-training is needed in case of new instances, for example new chemical compounds acting as drug-candidates. Moreover, storing the feature vectors of the training instances is also not necessary.

## Conclusion & Future Work

In this paper we have proposed a new tree-ensemble learning method, namely bi-clustering tree ensembles, for inferring interaction networks. The proposed approach is based on multi-label classification exploiting the multi-label structure of the interaction matrix, both in the part of tree-building and labeling. We performed a thorough evaluation study comparing our method to its direct tree-ensemble competitors. We validated the performance of our method in different interaction prediction settings and the obtained results affirmed its merits. The potential of our approach was reaffirmed by successfully revealing non-reported links in a previous version of a compound protein association network. Conclusively, the proposed method should be considered in network inference tasks, especially where interpretable models are desired.

An interesting topic for future research would be to build our approach on other tree-ensemble mechanisms and perform relevant comparisons. A comparison study between the bi-clustering inferred by our method and state of the art bi-clustering methods would be also an interesting topic of future research. In the future, the presented learning method should also be applied to large scale networks, performing this way *in silico* predictions which could be subsequently validated in the lab.

## Supplementary information

**Additional file 1** This file provides further information on our top reported associations from the STITCH dataset.

## Data Availability

The datasets used in this study are benchmark datasets and are publicly available. http://www.montefiore.ulg.ac.be/~schrynemackers/datasets

## Abbreviations

AUPR: Area under precision recall curve
AUROC: Area under the receiver operating characteristic curve
CPI: Chemical–protein interaction
DPI: Drug-protein interaction
DTI: drug-target interaction
E: Enzymes
eBICT: Ensemble of bi-clustering trees
ERN: E. coli regulatory network
ERT: Extremely randomized trees
GLSO: Global single output
GR: G-protein-coupled receptors
GRN: Gene Regulatory Networks
IC: Ion channels
LOCMO: Local multiple-output
MLkNN: Multi-label k-nearest neighbor
NR: Nuclear receptors
RF: Random forests
SRN: S. cerevisiae regulatory network
STC: Super target clustering
