# Supplementary material for: Network inference with ensembles of bi-clustering trees
Source: BMC Bioinformatics. 2019 Oct 28;20:525. doi: 10.1186/s12859-019-3104-y (PMC6819564; doi:10.1186/s12859-019-3104-y)
Supplement: Supplementary file 1 — Additional file 1 This file provides further information on our top reported associations from the STITCH dataset. [file 12859_2019_3104_MOESM1_ESM.pdf]

Supplemental Material

Network inference with ensembles of bi-clustering trees

Konstantinos Pliakos and Celine Vens

In our paper "Network inference with ensembles of bi-clustering trees" we investigated the performance of our proposed method (eBICT) by extracting a subset of the chemical compound association database STITCH. More specifically, we employed the specific dataset in two versions. The first derives from STITCH v3.1 and the second from STITCH v4. There are many links in the compound protein network that are not reported in v3.1 but exist in v4. We trained our method using the interaction matrix that corresponds to v3.1 and evaluated the predictions using the matrix of v4. The purpose of this experiment was to investigate whether the application of the proposed learning approach and more specifically the inferred bi-clustering could reveal not-yet-reported associations between existing nodes of a network.

Below there is a table presenting the top 20 associations identified by our proposed approach eBICT. These associations were not present in version 3.1 which was used to train our model but appear in version 4. The scores stored in STITCH stem from lab experiments, information from manually curated databases and computational approaches such as text mining. Thus, it is deducted that not all of those predicted associations can be translated as molecular interactions. This way we refer to the predicted links as associations instead of interactions.

Clearly, it can be noted that in most cases the predicted pair involves the protein Calmodulin and particularly calmodulin-3 which is a calmodulin protein encoded by gene CALM-3. The genes CALM-1, CALM-2, and CALM-3, are different and are found on different chromosomes (14, 2, and 19). However, they encode proteins, namely Calmodulin-1, calmodulin-2 and calmodulin-3, that have exactly the same 149-length amino acid sequence (primary structure). Thus, although post-translation processes could slightly alter the 3D structure of those proteins, associations between chemical compounds and calmodulin-1, or calmodulin-2 are likely to also appear between those compounds and calmodulin-3.

Chemical compound	protein	Score in v3.1	Score in v4	prediction scores
CID100000312 (chloride)	ENSP00000291295 (CALM3)	0	0.338	0.653
CID100000702 (ethanol)	ENSP00000291295 (CALM3)	0	0.330	0.653
CID100000710 (flavin mononucleotide)	ENSP00000291295 (CALM3)	0	0.258	0.654
CID100000813 (potassium)	ENSP00000272298 (CALM2)	0	0.453	0.650
CID100000888 (magnesium)	ENSP00000291295 (CALM3)	0	0.288	0.653
CID100000934 (nickel)	ENSP00000291295 (CALM3)	0	0.180	0.660

CID100001003 (phosphate)	ENSP00000291295 (CALM3)	0	0.472	0.664
CID100001023 (pyrophosphate)	ENSP00000291295 (CALM3)	0	0.181	0.651
CID100002533 (calphostin C)	ENSP00000291295 (CALM3)	0	0.260	0.653
CID100002551 (carbachol)	ENSP00000291295 (CALM3)	0	0.338	0.655
CID100002726 (chlorpromazine)	ENSP00000291295 (CALM3)	0	0.646	0.660
CID100002818 (clozapine)	ENSP00000291295 (CALM3)	0	0.814	0.654
CID100003559 (haloperidol)	ENSP00000291295 (CALM3)	0	0.831	0.653
CID100004584 (okadaic acid)	ENSP00000291295 (CALM3)	0	0.332	0.659
CID100005566 (trifluoperazine)	ENSP00000291295 (CALM3)	0	0.907	0.656
CID100011005 (myristate)	ENSP00000291295 (CALM3)	0	0.831	0.653
CID100045469 (sodium nitroprusside)	ENSP00000291295 (CALM3)	0	0.286	0.662
CID100083513 (barium)	ENSP00000291295 (CALM3)	0	0.834	0.650
CID100104751 (BAPTA)	ENSP00000291295 (CALM3)	0	0.399	0.663
CID100439177 (glycogen)	ENSP00000349467 (CALM1)	0	0.271	0.656
